# The endogenous glutamatergic transmitter system promotes collagen synthesis in cardiac fibroblasts under hypoxia

**DOI:** 10.3389/fcvm.2025.1638650

**Published:** 2025-10-17

**Authors:** Ruiyu Wang, Xiao Yuan, Ting Xie, Xin Zhao, Junying Li, Xiaohong Zhang, Chuanzhu Lv

**Affiliations:** ^1^ Emergency Medicine Center, Sichuan Provincial People’s Hospital, University of Electronic Science and Technology of China, Chengdu, China; ^2^ Department of Cardiovascular Medicine, Cardiovascular Research Center, The First Affiliated Hospital of Chongqing Medical University, Chongqing, China

**Keywords:** L-glutamate, collagen production, cardiac fibroblasts, myocardial fibrosis, hypoxia

## Abstract

Excessive collagen production is a hallmark of cardiac fibroblasts (CFs) activation and plays a pivotal role in the pathogenesis of myocardial fibrosis (MF). Hypoxia, a key pathogenic factor in MF, induces aberrant biological responses in CFs and is closely associated with CFs activation. This study investigates the mechanisms underlying hypoxia-induced fibrogenesis from a metabolomics perspective. Neonatal rat CFs were isolated and cultured under normoxic or hypoxic conditions. Hypoxia significantly increased collagen production in CFs, as indicated by the upregulation of Collagen I and Collagen III expression. Non-targeted metabolomics profiling revealed significant alterations in the secretory metabolites of CFs under hypoxia, among which, L-glutamate levels were markedly elevated. Furthermore, L-glutamate concentrations were significantly increased in the myocardial tissues of rats with myocardial infarction. Key components of the glutamatergic transmitter system, including glutamate receptors, metabolic enzymes, and transporters, were detected in CFs, and their expression was upregulated under hypoxic conditions. Notably, exogenous L-glutamate supplementation promoted collagen production in CFs even under normoxia. Blocking glutamate receptors with CNQX and MK-801 effectively reversed hypoxia-induced increases in Collagen I and Collagen III protein expression. Additionally, both CNQX and MK-801 significantly downregulated TGF-*β* protein expression and Smad2/3 phosphorylation in hypoxia-stimulated CFs. These findings demonstrate that L-glutamate mediates collagen production in CFs under hypoxia, partially through activation of the TGF-*β*/Smad signaling pathway. Targeting the glutamatergic transmitter system may offer a novel therapeutic strategy for MF.

## Introduction

Hypoxia is one of the major pathogenic factors contributing to myocardial injury during myocardial infarction (MI). The hypoxia-derived activation of cardiac fibroblasts (CFs) has been identified as a key mediator in the process of wound healing following infarction and plays a significant role in adverse myocardial remodeling (1). Upon activation, CFs typically transform to a myofibroblast phenotype and secrete extracellular matrix (ECM) components to facilitate myocardial repair. However, excessive and uncontrolled ECM deposition, characterized by the overproduction of collagen in the myocardium, can disrupt myocardial architecture, exacerbate cardiac vasoconstriction, and ultimately lead to myocardial fibrosis (MF) and heart failure (2). CFs are highly sensitive to ischemia and hypoxic conditions, and can secrete a variety of bioactive compounds, including inflammatory and profibrotic cytokines, exosomes, and matrix metalloproteinases, which collectively modulate the progression of MF (3–6). Therefore, elucidating the mechanisms of intercellular communication among CFs within the myocardial microenvironment has emerged as a crucial research direction for uncovering the underlying pathogenesis of MF.

Metabolomics provides a powerful and efficient tool for uncovering metabolic signatures and alterations at both the local and systemic levels, primarily through advanced analytical techniques such as mass spectrometry (MS) and nuclear magnetic resonance (NMR) spectroscopy (7). In the context of MF, metabolomics profiling has been extensively applied across various diagnostic and therapeutic domains, particularly in biomarker discovery, disease progression monitoring, and identifying novel therapeutic targets (8). Hypoxia induces substantial alterations in the synthesis and secretion of various bioactive metabolites, including amino acids, lipids, and nucleotides, which can exert direct or indirect regulatory effects on cellular function (9). Although the heart undergoes adaptive metabolic responses to enhance its tolerance to reduced oxygen availability under hypoxia, these compensatory changes ultimately lead to impaired cardiac function in patients with chronic hypoxia (10). Research has revealed significant mitochondrial dysfunction and oxidative stress in CFs under hypoxic conditions, which may be closely associated with the development of MF (11, 12). However, the precise molecular mechanisms underlying hypoxia-induced CFs activation remain to be fully elucidated.

L-glutamate, also known as glutamate, is one of the most abundant excitatory neurotransmitters in central nervous system. It plays a crucial role in delivering electrical excitation between neurons through evoking excitatory postsynaptic currents (13). Extracellular glutamate can bind to ionotropic glutamate receptors (iGluRs) and metabotropic glutamate receptors (mGluRs), thereby modulating intracellular signal transduction (14). Dysregulation of glutamate or glutamate receptor activity has been linked to multiple neuropsychiatric disorders, including neurodegenerative diseases, epilepsy, schizophrenia and mood disorders (15, 16). Consequently, maintaining optimal glutamate balance is a crucial therapeutic target for neuropsychiatric conditions. Emerging research has indicated that, in addition to its essential functions in nervous system, glutamate exerts significant regulatory effects on cardiovascular system (17). Although glutamine supplementation demonstrated protective effects against ischemia-reperfusion and isoprenaline-induced myocardial injury (18, 19), elevated plasma glutamate levels have been associated with increased risk of heart failure in diabetic patients (20), underscoring the complex and multifunctional biological properties of glutamate on cardiac function. Notably, a recent study has identified an endogenous glutamatergic transmitter in atrial cardiomyocytes, which can directly modulate the generation of cardiac action potential (21). Despite these advancements, the expression and regulatory effects of glutamatergic transmitter system on CFs remain unexplored.

Due to its polar and water-soluble properties, L-glutamate can be accurately identified and quantified using modern analytical techniques. In this study, metabolomics profiling was initially employed to systematically characterize the changes in the secretory products of CFs under hypoxia. L-glutamate levels were detected in both the supernatant of CFs and the myocardial tissues of rats. Importantly, this study provided the first comprehensive identification of key components of glutamatergic transmitter system in CFs. Furthermore, the regulatory effects and potential mechanisms of L-glutamate in hypoxia-induced CFs activation were further elucidated.

## Materials and methods

### Establishment of MI rat model

Twenty male Sprague–Dawley (SD) rats (8 weeks old) were obtained from Chengdu Dashuo Laboratory Animal Co., Ltd. in Chengdu, China (certificate No. SYXK-2018-110) and maintained under specific pathogen-free conditions. All animal procedures were conducted in accordance with the Ethics Committee of Sichuan Provincial People's Hospital (Approval No. 2023-0364) and complied with the Guidelines for the Care and Use of Laboratory Animals published by the National Institutes of Health.

The MI model was established following our previously described method (22). Briefly, the rats were anesthetized using isoflurane and intubated with a tracheal cannula. A left thoracotomy was performed between the fourth and fifth ribs on the left chest wall. Mechanical ventilation was then performed using oxygen supplemented with 1.5% isoflurane. A standard lead II electrocardiogram (ECG) system was subcutaneously attached to the rat's limbs using needle electrodes. The left anterior descending (LAD) coronary artery was carefully visualized and ligated using an 8–0 prolene suture. Sham-operated rats underwent same surgical procedures without LAD ligation. Successful induction of acute MI was confirmed by real-time ECG monitoring, evidenced by ST-segment elevation. All the rats were sacrificed 12 h post-surgery, and myocardial tissues were harvested for subsequent analysis.

### Triphenyltetrazolium chloride (TTC) staining of myocardial tissues

TTC staining was performed to visualize viable and infarcted myocardium in cardiac tissues. Briefly, fresh myocardial tissues were obtained from sham-operated and MI rats 12 h after surgery and transversely sectioned into 2 mm thick slices using a precision tissue slicer. Subsequently, the tissue sections were immersed in 1% TTC solution (Solaibao Biotechnology, Beijing, China) and incubated at 37°C for 20 min. The images of sections were photographed using a high-resolution digital camera.

### Isolation and culture of neonatal rat CFs

Neonatal rat CFs were isolated from SD rats within 7 days of birth using a modified enzymatic digestion protocol as previously described (22). Briefly, neonatal rat hearts were rapidly excised and minced into small tissue fragments. The tissue was then digested in an enzymatic solution containing 0.1% collagenase type II (Mengbio, Chongqing, China) and 0.08% trypsin (Beyotime, Shanghai, China) at 37°C for 30 min. Following digestion, the cell suspension was centrifuged at 1,000 rpm for 5 min, and the resulting pellet was resuspended in Gibco™ BASIC DMEM (containing 4.5 g/L D-glucose, 10 mM L-glutamine, and 110 mg/L sodium pyruvate), supplemented with 10% fetal bovine serum (FBS; OriCell, USA). The cells were cultured in a humidified incubator at 37°C with 5% CO_2_. CFs were purified using a differential adhesion method. Specifically, the cell suspension was incubated for 1 h to allow CFs to adhere, after which non-adherent cells (most are cardiomyocytes) were removed. The adherent CFs were replenished with fresh medium and maintained under standard culture conditions. Cells at the first passage were used for subsequent experiments.

### Hypoxia induction and treatment of CFs

To eliminate the potential interference of serum components on metabolomic profiling results, the culture medium of CFs was replaced with FBS-free DMEM prior to hypoxia induction. Subsequently, the cells were transferred to a hypoxic chamber maintained at 1% O_2_, 5% CO_2_, and 94% N_2_. To confirm the successful establishment of hypoxic conditions, CFs were pre-incubated with Image-IT™ Green Hypoxia Reagent (ThermoFisher, Germany), a highly sensitive hypoxia detection probe, before exposure to hypoxia. The hypoxia-induced green fluorescence signals were monitored and captured at predetermined time intervals using a fluorescence microscope (Carl Zeiss, Germany).

To specifically inhibit iGluRs, CFs were pretreated with two antagonists: CNQX (an AMPA/kainate receptor antagonist; from MedChemExpress, USA) and MK-801 (a NMDA receptor antagonist; from MedChemExpress, USA), both at a concentration of 100 μM. The dosages of CNQX and MK-801 were determined based on findings from a previous study (21). After 12 h of hypoxia treatment, CFs were collected for subsequent experiments.

### Cell immunofluorescence

CFs were fixed with 4% paraformaldehyde and permeabilized using 0.25% Triton X-100. To block non-specific binding sites, the cells were treated with 1% bovine serum albumin (BSA) at room temperature for 30 min. Subsequently, the cells were incubated overnight at 4°C with primary antibodies against Vimentin (1:100; Proteintech, China), Collagen I (1:200; Proteintech, China), Collagen III (1:200; Proteintech, China), *α*-SMA (1:200; Proteintech, China), and p-Smad2/3 (1:100; Affinity, China). After washing, the cells were incubated with Cy3- or FITC-conjugated secondary antibodies (1:500; Proteintech, China) for 1 h at room temperature. Nuclei were stained using DAPI solution (Beyotime, China). Fluorescence signals were captured using a fluorescence microscope (Carl Zeiss, Germany), and quantitative analysis was conducted using Image-Pro Plus software.

### Intracellular calcium ion detection

Intracellular calcium levels were detected using the fluorescent dye Fluo-4AM (Beyotime, China). Briefly, CFs were incubated with Fluo-4 AM solution at 37℃ for 30 min. Following incubation, the cells were washed three times with PBS. Fluorescence signals were captured using a fluorescence microscope (Carl Zeiss, Germany), and quantitative analysis was performed using Image-Pro Plus software.

### Non-targeted metabolomics profiling

Non-targeted metabolomics profiling was conducted on the supernatant of CFs under both normoxic and hypoxic conditions. Following sample pretreatment, metabolites were extracted from the supernatant and subjected to analysis using a liquid chromatography-tandem mass spectrometry (LC-MS) system (ThermoFisher, Germany) coupled with an Orbitrap Q ExactiveTMHF-X mass spectrometer (Thermo Fisher, Germany) at Novogene Co., Ltd. (Beijing, China). The raw data obtained from LC-MS were processed using Compound Discoverer 3.3 (CD3.3, ThermoFisher) for peak alignment, peak picking, and metabolite quantitation. Differential expression analysis was conducted using the DESeq2 R package. Metabolites with a VIP > 1, *P*-value < 0.05, and a fold change (FC) ≥ 2 or FC ≤ 0.5 were identified as differential metabolites. Functional enrichment of these metabolites was based on the KEGG database, with a *P*-value < 0.05 considered statistically significant for enrichment.

### Enzyme-linked immunosorbent assay (ELISA)

Myocardial tissue samples were obtained from sham-operated rats (normal tissue) and the infarct border zones of MI rats. After weighing, the tissues were thoroughly homogenized and diluted with phosphate buffer. Subsequently, the homogenates were centrifuged at 1,000 × g for 10 min at 4°C. The concentration of L-glutamate in both the cell culture medium of CFs and myocardial tissue was quantitatively measured using an ELISA assay kit (ZCi Bio; Shanghai, China) according to the manufacturer's instructions.

### Agarose gel electrophoresis and real-time polymerase chain reaction (RT–PCR)

Total RNA was extracted from CFs using TransZol Up reagent (TransGen, Beijing, China). The RNA was then reverse-transcribed into cDNA using Uni One-Step gDNA Removal and cDNA Synthesis SuperMix (TransGen Beijing, China). Total cDNA was amplified using One-Step qRT–PCR SuperMix (TransGen Beijing, China). RT–PCR was performed on a QuantStudio real-time system (ThermoFisher, Carlsbad, USA) under the following conditions: initial denaturation at 95℃ for 1 min, followed by 40 cycles of denaturation at 95℃ for 10 s and annealing/elongation at 60℃ for 30 s. The original Ct values were standardized to α-tubulin using the 2^− ΔΔCt^ method. The primer sequences were provided in Table 1.

**Table 1:** Sequences of primers used in RT-PCR.

Gene (Rat)	Oligonucleotide primer sequences (5′–3′)
*α*-tubulin	Forward: TGCCAAGCGACAAGACCAT
	Reverse: GCCAGTGCGAACTTCATCAAT
SLC1A3	Forward: TGGTAGCGGTGATAATGTGGTA
	Reverse: AGGAGAGGCAGGACGATGA
GRIA1	Forward: GGTGGTGGTTGACTGTGAATC
	Reverse: CATTGGCTCCGCTCTCCTT
GRIA2	Forward: TTGGAATGGTATGGTTGGAGAG
	Reverse: TGGACTTCTGAGGCTTCTTGA
GRIA3	Forward: TGTGGCAGGCGTGTTCTATA
	Reverse: TGAGTGTTGGTGGCAGGAG
GRIA4	Forward: AACTGTGTTGGTGACTGACTG
	Reverse: AGTATGCTCTTCCTGCTCTCAA
GRIN1	Forward: GCACCACTGACCATCAACAAT
	Reverse: AGCATCACAGCCACCACAT
GRIN2	Forward: TCAAGACAACAGTGGACAACAG
	Reverse: GAGCAGGATGACAGAAGAATGG
GRIN3	Forward: CTCTCCATCCTGACCACCATT
	Reverse: TCCATCTTCTCCATCTGCTCTT
GRIK1	Forward: ATATCGGCGGCATCTTCATTG
	Reverse: ACTTCTCTACACCAAGGCTCTC
GRIK2	Forward: CGACTGATGCTGCTCTGATG
	Reverse: TTCTGCCTGTGAGACCTTCC
GRIK3	Forward: TCTGAGGTGGTGGAGAATAACTT
	Reverse: GCCGTGTAGGAGGAGATGATAA
GRIK4	Forward: TGCTTCCTGCTTGGCTCTT
	Reverse: CGGTTGATGCGGTTCTTGG
GRIK5	Forward: TGCCTGCTGCGGTTAGAA
	Reverse: CGATGATGATGGTGGACACTT
GLS	Forward: GCAGTCAGCGGACATACCA
	Reverse: AACCAAGCCACAGAGACAGTA

To analyze gene expression related to the glutamatergic transmitter system in CFs, the RT-PCR products derived from normal CFs were subjected to agarose gel electrophoresis. The samples were run at 120 V for 40 min. Subsequently, the gel was imaged using a multifunctional gel imaging system (Tanon, China) to visualize the DNA bands.

### Cell activity detection

The cell viability assay was performed using the CCK-8 method. Briefly, CFs were reseeded in 96-well plates and exposed to varying concentrations of L-glutamate (MedChemExpress, USA) ranging from 0.1 to 5 mM for 12 h, then the supernatant was removed and replaced with fresh culture medium. Subsequently, 100 μl of DMEM containing 10 μl of CCK8 solution (MedChemExpress, USA) was added to each well. The plates were then incubated at 37°C for 1 h, after which the optical density (OD) at 450 nm was measured using a microplate spectrophotometer (Allsheng, China).

### Western blot studies

Total protein was extracted from myocardial tissues of rats and CFs using RIPA lysis buffer. Protein samples were separated by SDS‒PAGE and transferred onto PVDF membranes. The membranes were blocked with 5% BSA for 1.5 h at room temperature, followed by overnight incubation at 4°C with the following primary antibodies: Collagen I (1:2,000; Proteintech), Collagen III (1:2,000; Proteintech), α-SMA (1:3,000; Proteintech), GRIN2 (1:1,000; Proteintech), GRIA3 (1:1,000; Proteintech), SLC1A3 (1:1,000; HuaBio), GLS (1:1,000; HuaBio), TGF-β (1:1,000; Proteintech), Smad2/3 (1:1,000; Affinity, China), p-Smad2/3 (1:100; Affinity, China) and α-tubulin (1:3,000; Proteintech). After incubation with primary antibody, the membranes were treated with HRP-conjugated secondary antibodies (1:5,000; Proteintech) for 1 h at room temperature. Protein bands were visualized using an enhanced ECL kit (Beyotime, China). α-Tubulin served as the internal control. Where necessary, membranes were stripped and re-probed with the appropriate antibodies. Quantitative analysis of band intensities was performed using Image-Pro Plus software.

### Statistical analyses

The statistical analyses were conducted using SPSS 20.0 software (IBM Corporation, USA). All results were expressed as mean ± standard deviation (SD). The comparison between two groups was conducted using the Student's *t* test. One-way ANOVA followed by Tukey's *post hoc* test was used to assess the statistical differences among more than two groups. A *P*-value < 0.05 was considered statistically significant for all statistical tests.

## Results

### Hypoxia stimulated collagen synthesis in CFs

**Figure 1:**
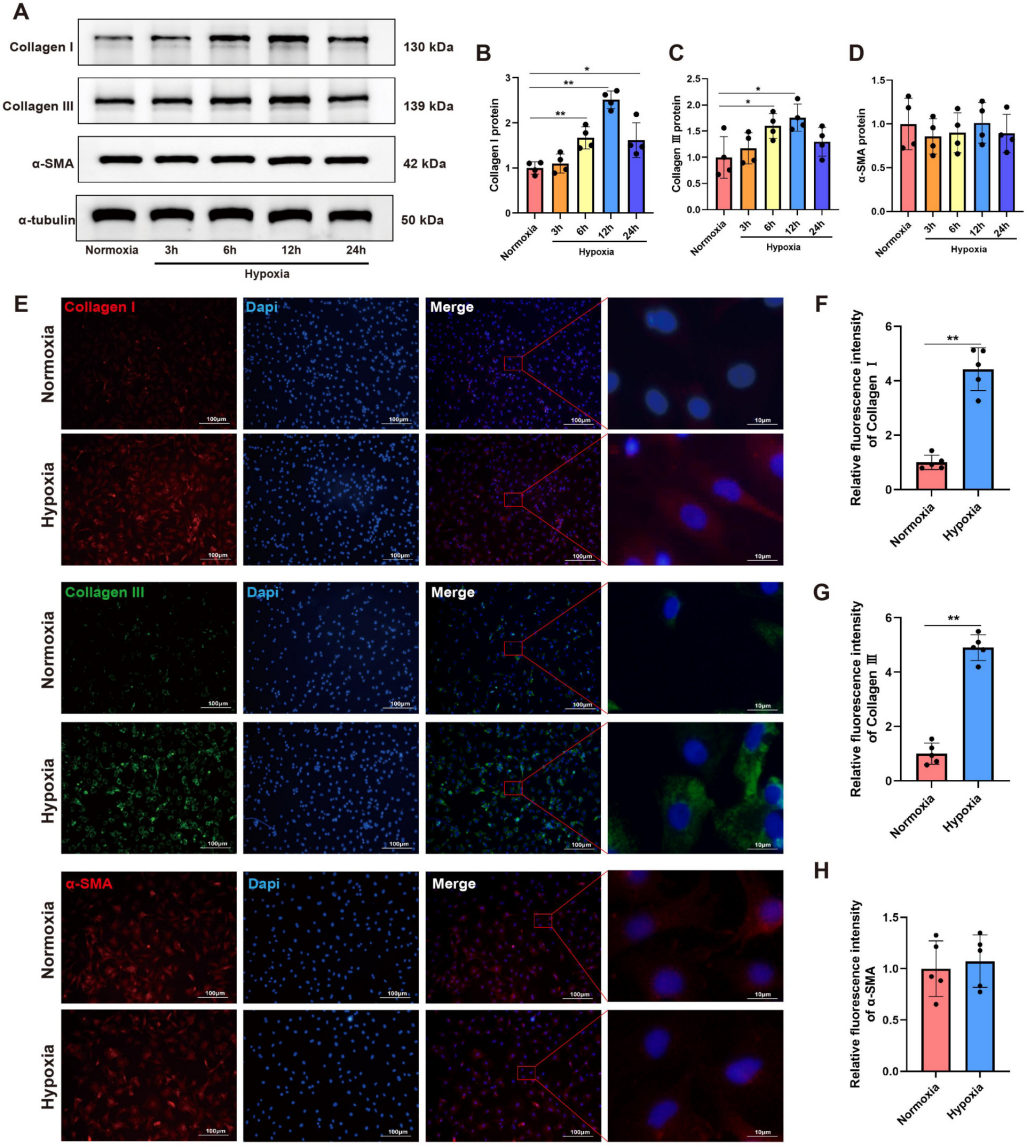
Hypoxia stimulated collagen synthesis in CFs. **(A)** Western blots showing the protein levels of Collagen I, Collagen III and α-SMA in CFs. **(B–D)** Quantitative analysis of Collagen I **(B)**, Collagen III **(C)** and *α*-SMA **(D)** protein expression in each group. **(E)** Immunofluorescence images showing the expression of Collagen I, Collagen III and *α*-SMA in CFs; Collagen I and α-SMA were labeled with red fluorescence, while Collagen III was labeled with green fluorescence; original magnification ×100 and ×1,000. **(F–H)** Quantitative analysis of the fluorescence intensity of Collagen I **(F)**, Collagen III **(G)** and α-SMA **(H)** expressions in each group. *P < 0.05 vs. normoxia group; **P < 0.01 vs. normoxia group.

The dynamic growth of newly isolated CFs at different time points was presented in Supplementary Figure S1A. For cell identification, immunofluorescence staining was performed on isolated cells using vimentin, a specific biomarker for fibrocytes (Supplementary Figure S1B). To investigate the impact of hypoxia on CFs activation, we initially introduced a sensitive hypoxia detection probe to visualize hypoxic regions during different durations of hypoxia exposure. Fluorescence imaging of CFs, along with the corresponding bright-field and merged images, were shown in Supplementary Figure S2. A detectable fluorescence signal emerged 3 h post-hypoxia treatment, with the signal intensity progressively increasing over time. After 12 h of hypoxia exposure, notable morphological changes in CFs were observed, including cell elongation, spindle-shaped morphology, and excessive ECM deposition surrounding the cells. On this basis, Western blot analysis demonstrated that the protein levels of both Collagen I and Collagen III were gradually upregulated, and their expressions peaked at 12 h of hypoxia exposure (Figures 1A–C). Notably, hypoxia did not significantly alter α-SMA expression in CFs (Figure 1D). To further characterize the expression and localization of these proteins, immunofluorescence staining was performed on CFs subjected to 12 h of hypoxia exposure. As shown in Figures 1E–H, hypoxia significantly increased the fluorescence intensity of both Collagen I and Collagen III compared to normoxic conditions. Consistent with Western blot findings, hypoxia did not induce substantial changes in α-SMA expression in CFs. These findings indicated that hypoxia could promote CFs activation, particularly in stimulating collagen synthesis, but did not significantly influence their phenotypic transformation into myofibroblasts, as indicated by the unchanged α-SMA expression.

### Non-targeted metabolomics profiling revealed upregulation of L-glutamate levels in the secretions of hypoxia-induced CFs

**Figure 2:**
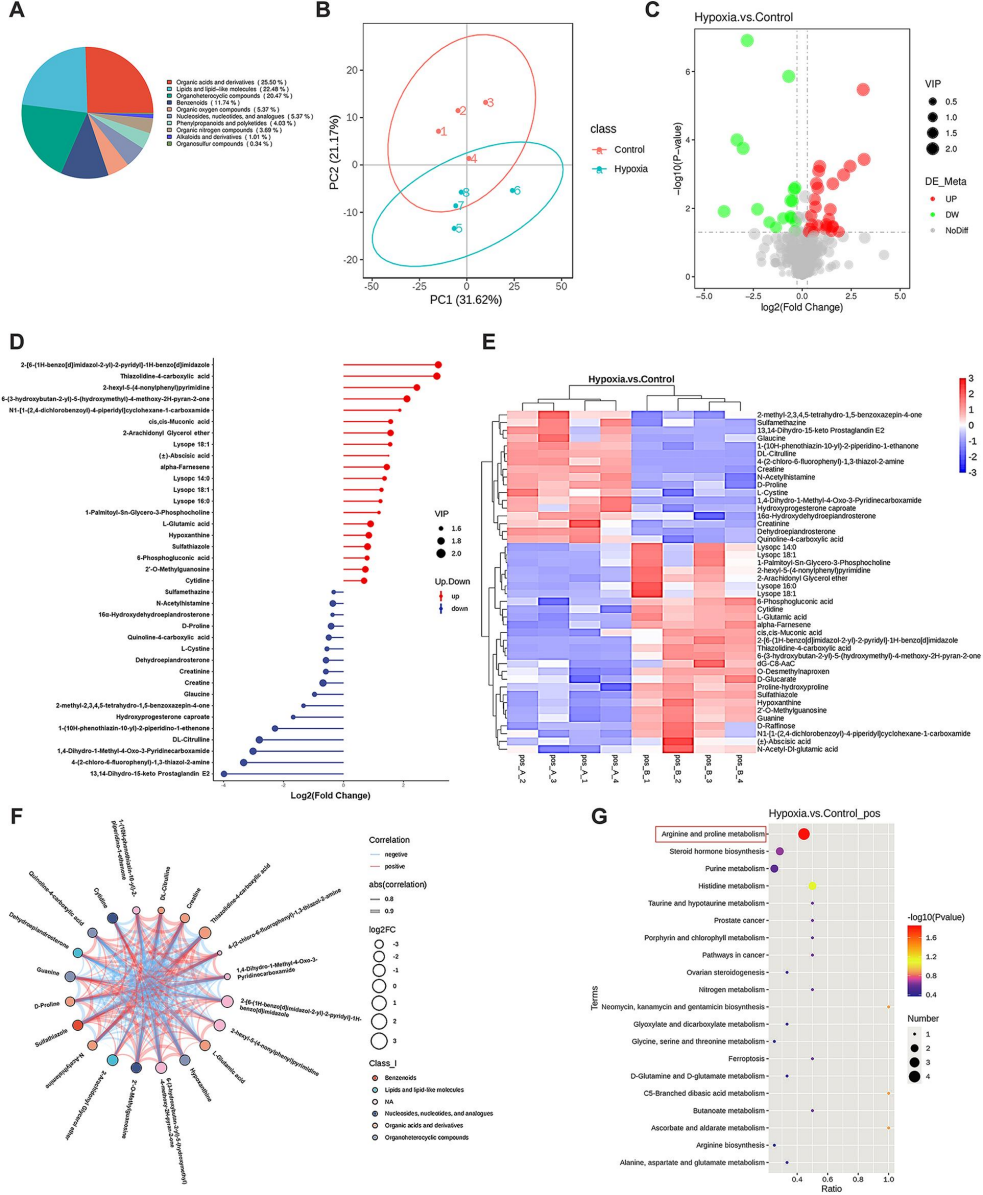
Non-targeted metabolomics analysis of CF secretions under different oxygen conditions. CFs were cultured under either normoxic or hypoxic conditions for 12 h, then the cell culture medium supernatants were collected for metabolomics profiling. **(A)** Pie chart of metabolite classification (Class I). **(B)** Principal component analysis demonstrating the metabolic profile variations between groups. **(C)** Volcano plot illustrating the differentially expressed metabolites, with statistical significance and fold-change thresholds indicated. **(D)** Matchstick map highlighting the top 20 most significantly altered metabolites based on their quantitative changes. **(E)** Hierarchical clustering analysis of differentially expressed metabolites, showing distinct patterns between normoxic and hypoxic conditions. **(F)** Chord diagram displaying the correlations among the top 20 differential metabolites. **(G)** KEGG pathway enrichment analysis of hypoxia-induced differential metabolites.

**Figure 3:**
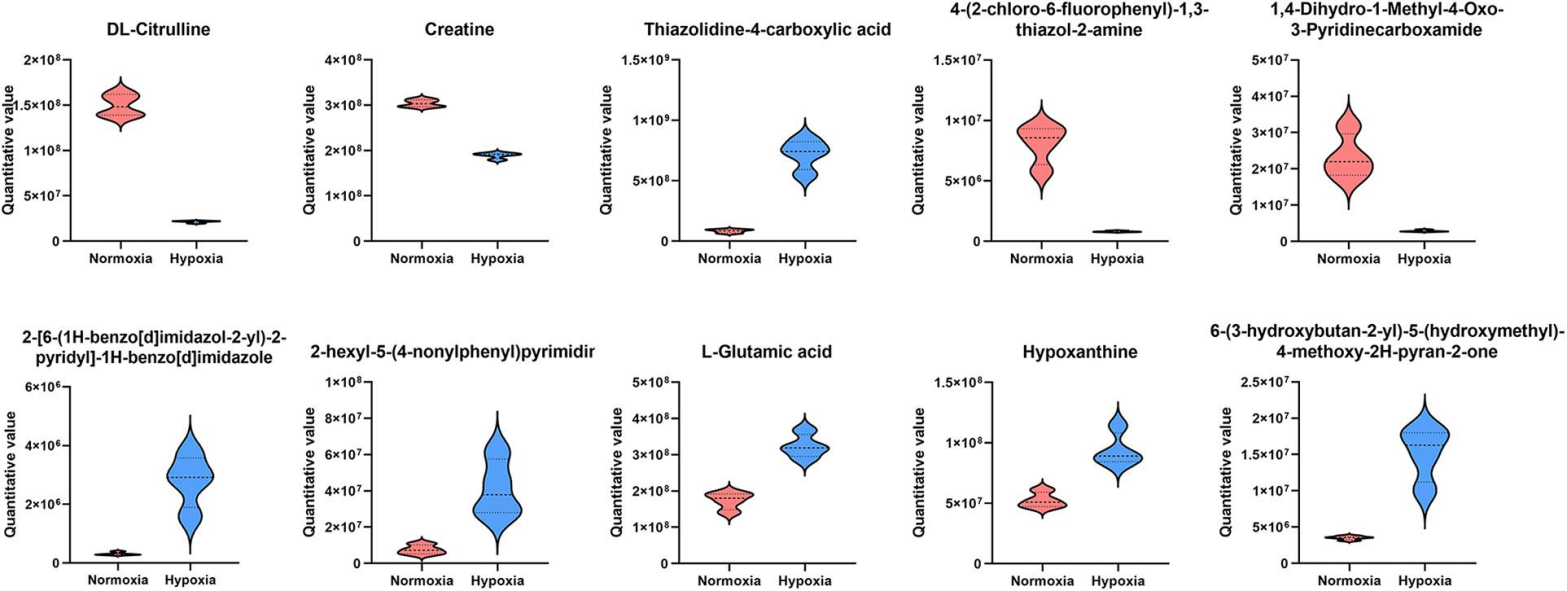
The top 10 most significantly altered metabolites in the secretions of hypoxia-stimulated CFs.

To investigate hypoxia-induced metabolic alterations, secreted products from CFs cultured under normoxic and hypoxic conditions were collected for non-targeted metabolomics analysis (*n* = 4 per group). The identified metabolites were classified by chemical category, with the majority belonging to organic acids and derivatives (25.50%) and organoheterocyclic compounds (20.47%) (Figure 2A). Principal component analysis (PCA) demonstrated favorable methodological stability and high data quality (Figure 2B). Metabolomics profiling identified 44 differentially expressed metabolites between the control (normoxia) and hypoxia groups, with 27 metabolites upregulated and 17 downregulated in the hypoxia group, as shown in the volcano plot (Figure 2C). The top 20 most significantly altered metabolites, along with their quantitative levels, were visualized using a matchstick map (Figure 2D). Hierarchical clustering analysis revealed distinct metabolic profiles between the two groups, with L-glutamate levels showing significant upregulation in the hypoxia group (Figure 2E). Further correlation analysis among the top 20 differential metabolites was shown in a chord diagram display (Figure 2F), indicating that L-glutamate positively correlated with 2-hexyl-5-(4-nonylphenyl) pyrimidine and 2-Arachidonyl Glycerol ether, while negatively correlated with N-Acetylhistamine and 4-(2-chloro-6-fluorophenyl)-1,3-thiazol-2-amine. KEGG pathway analysis revealed significant enrichment of hypoxia-induced differential metabolites in the arginine and proline metabolism pathways (Figure 2G). The top 10 most significantly altered metabolites, including their chemical names and quantitative analysis (all *P* values < 0.001), was systematically illustrated in Figure 3. These findings demonstrated that hypoxia induced substantial metabolic alterations in CF secretions, particularly highlighting the upregulation of L-glutamate levels and activation of the arginine and proline metabolism signaling pathways in hypoxia-stimulated CFs.

### Activation of the glutamatergic transmitter system in hypoxia-induced CFs and myocardial tissues of MI rats

**Figure 4:**
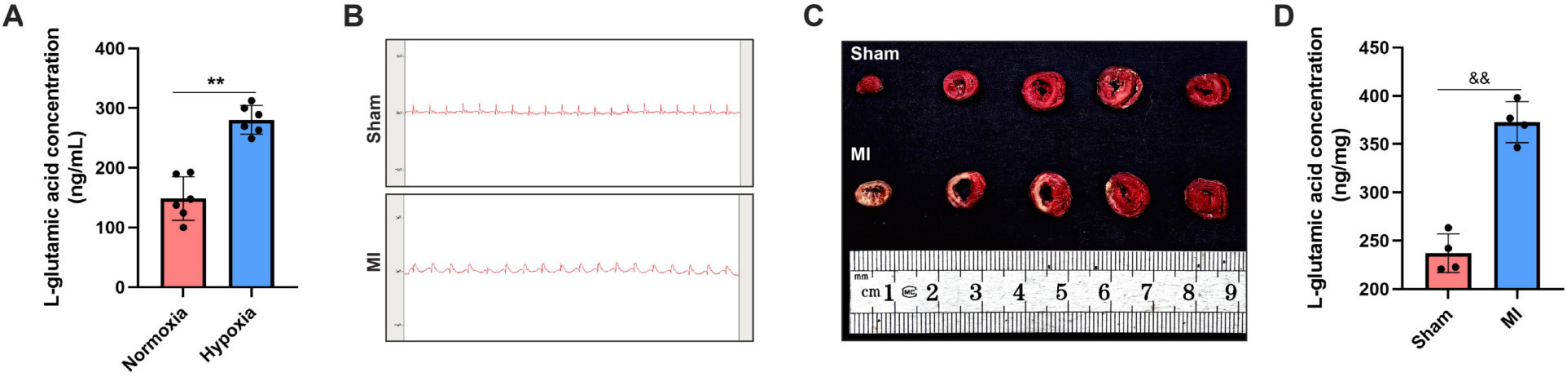
L-glutamate levels were elevated in both the secretory products of CFs and the myocardial tissues of MI rats. **(A)** ELISA assay of L-glutamate concentration in the culture medium of CFs. **(B)** ECG monitoring of rats in the sham and MI groups. **(C)** TTC staining demonstrating viable (red-stained) and infarcted (pale-stained) myocardial tissues in the sham and MI groups. **(D)** ELISA assay of L-glutamate concentration in the myocardial tissues of rats.^&&^*P* < 0.01 vs. sham group; *P < 0.05 vs. normoxia group; **P < 0.01 vs. normoxia group.

To validate the metabolomics profiling results, the culture medium of CFs under both normoxic and hypoxic conditions was collected for ELISA assay. The results demonstrated that the concentration of L-glutamate was significantly increased in the hypoxia group compared with the normoxia group (280.54 ng/ml vs. 148.71 ng/ml; *P* < 0.05; Figure 4A). In addition, we detected the alterations in L-glutamate levels in the myocardial tissues of rats. The successful establishment of MI animal model was confirmed by both ECG monitoring and TTC staining. Specifically, ECG showed an obvious elevation of ST-segment (Figure 4B), and TTC staining revealed pale or white staining in the infarcted myocardial tissues of MI rats (Figure 4C). On this basis, we detected L-glutamate concentrations and found that its level was significantly increased in the marginal area of infarcted myocardial tissues compared to the normal myocardial tissues (*P* < 0.05; Figure 4D).

**Figure 5:**
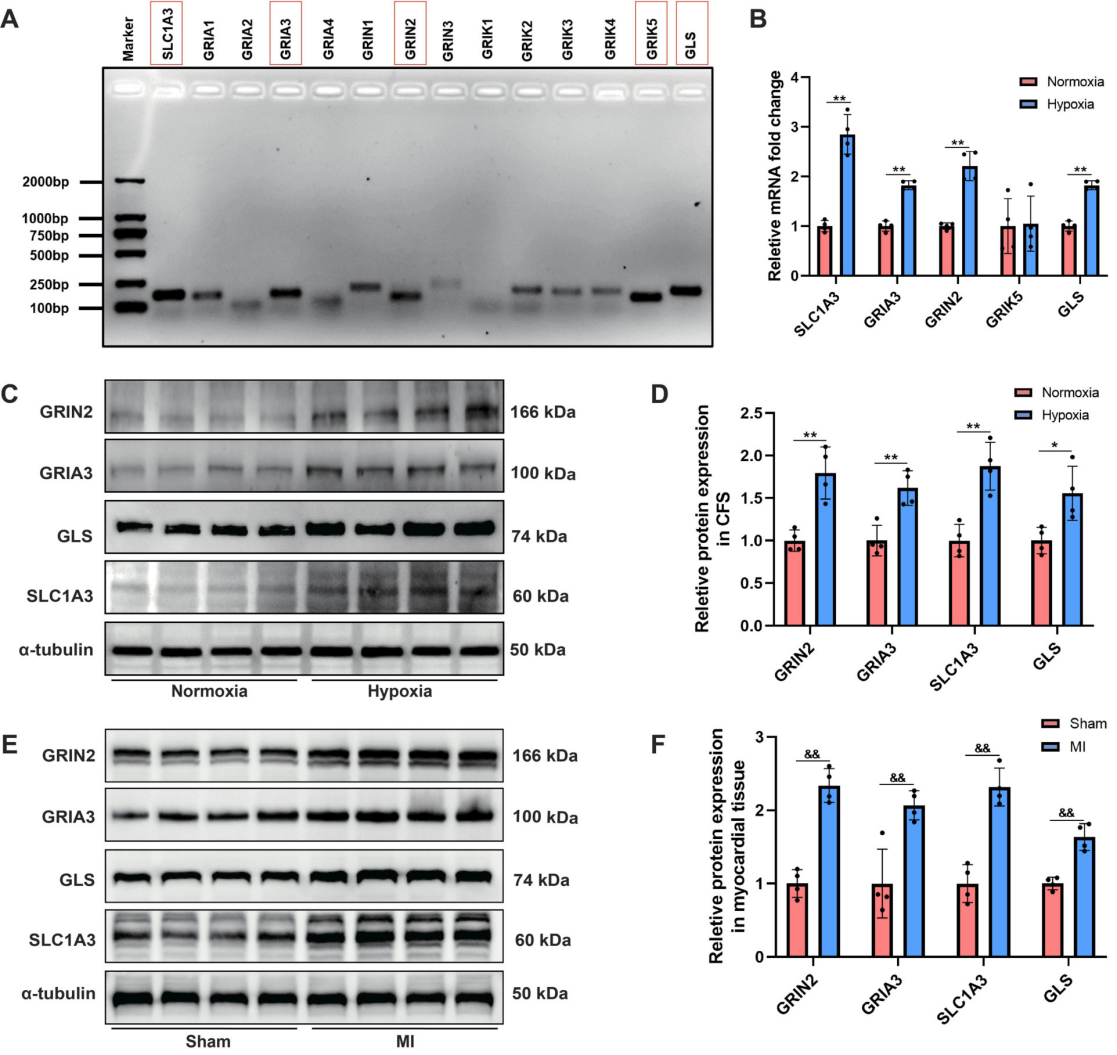
Activation of the glutamatergic transmitter system in hypoxia-induced CFs and myocardial tissues of MI rats. **(A)** Agarose gel electrophoresis displaying the major gene expression involved in the glutamatergic transmitter system in CFs. **(B)** RT–PCR analysis of the mRNA levels of SLC1A3, GRIA3, GRIN2 and GLS in CFs. **(C)** Western blots showing the protein levels of GRIN2, GRIA3, SLC1A3 and GLS in CFs. **(D)** Qquantitative analysis of GRIN2, GRIA3, SLC1A3 and GLS protein expression in CFs. **(E)** Western blots showing the protein levels of GRIN2, GRIA3, SLC1A3 and GLS in the myocardial tissues of rats. **(F)** Quantitative analysis of GRIN2, GRIA3, SLC1A3 and GLS protein expression in the myocardial tissues of rats. ^&&^*P* < 0.01 vs. sham group; *P < 0.05 vs. normoxia group; **P < 0.01 vs. normoxia group.

In addition to glutamate, the other major components in the glutamatergic transmitter system, including all subtypes of iGluRs (AMPA, NMDA and kainate receptors), the glutamate transporter protein (SLC1A3), and glutaminase (GLS) were detected. For the first time, we revealed that the following genes: SLC1A3, GRIA3, GRIN2, GRIK5 and GLS were relatively highly expressed in CFs (Figure 5A). RT–PCR analysis demonstrated that hypoxia induced significant upregulation of SLC1A3, GRIA3, GRIN2 and GLS mRNA levels in CFs, but did not affect GRIK5 expression (Figure 5B). Western blot analysis further revealed that the protein levels of GRIN2, GRIA3, GLS and SLC1A3 were upregulated in both the hypoxia-stimulated CFs and myocardial tissues of MI rats (Figures 5C–F). Collectively, these findings demonstrated that hypoxia induced activation of the glutamatergic transmitter system in CFs, which might serve as a crucial trigger mediating CFs activation.

### Exogenous supplementation of L-glutamate promoted collagen synthesis in CFs under normoxia

**Figure 6:**
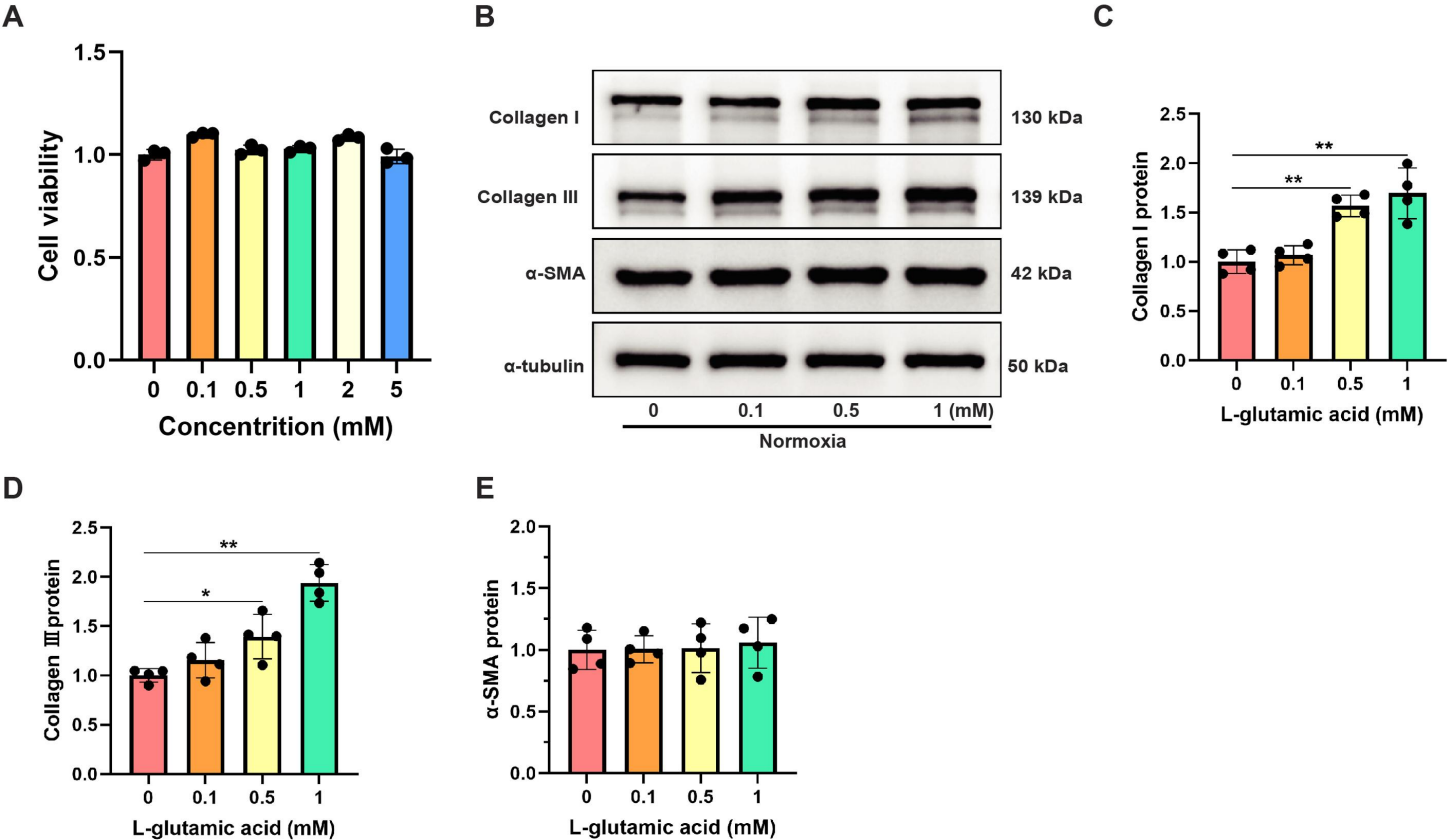
Exogenous supplementation of L-glutamate promoted collagen synthesis in CFs under normoxia. **(A)** Cell viability detection using CCK-8 assay in CFs treated with different L-glutamate concentrations. **(B)** Western blots showing the protein levels of Collagen I, Collagen III and α-SMA in CFs. **(C–E)** Quantitative analysis of Collagen I **(C)**, Collagen III **(D)** and α-SMA **(E)** protein expression in each group. *P < 0.05 vs. control group; **P < 0.01 vs. control group.

To investigate the specific regulatory effects of L-glutamate on CFs activation, CFs were exposed to varying concentrations of L-glutamate under normoxic conditions, and harvested for subsequent analyses 12 h post-treatment. Initially, CCK8 assay demonstrated that L-glutamate concentrations ranging from 0.1 to 5 mM had no significant impact on CFs viability (Figure 6A), suggesting no significant cytotoxicity of L-glutamate at these concentrations. Western blot analysis demonstrated that L-glutamate dose-dependently upregulated the protein levels of Collagen I and Collagen III in CFs. Although a slight increase in α-SMA expression was observed in CFs following L-glutamate treatment, its alternation was markedly less pronounced compared to the significant upregulation of Collagen I and Collagen III (Figures 6B–E). These findings confirmed that L-glutamate could effectively promote collagen synthesis in CFs without inducing substantial phenotypic transformation.

### L-glutamate activated the TGF-β/Smad signaling pathway in CFs under hypoxia

**Figure 7:**
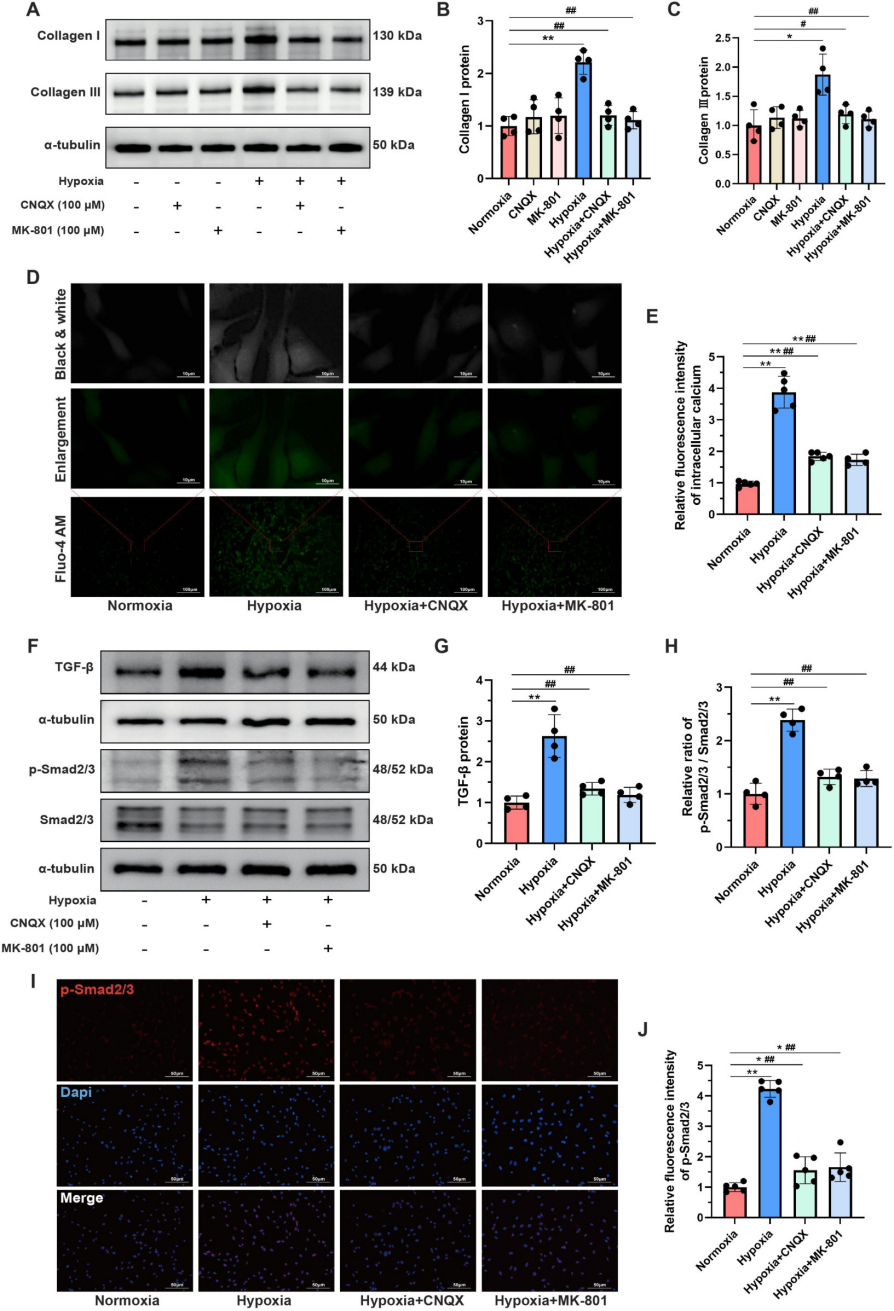
L-glutamate activated the TGF-β/Smad signaling pathway in CFs under hypoxia. **(A)** Western blots showing the protein levels of Collagen I and Collagen III in CFs. **(B,C)** Quantitative analysis of Collagen I **(B)** and Collagen III **(C)** in each group. **(D)** Detection of intracellular calcium ions using the fluorescent dye Fluo-4 AM; original magnifications: ×100 (lower panel) and ×1,000 (upper panel). **(E)** Quantitative analysis of the fluorescence intensity representing calcium levels in each group. **(F)** Western blots showing the protein levels of TGF-β, Smad2/3 and p-Smad2/3 in CFs. **(G,H)** Quantitative analysis of TGF-β protein expression **(G)** and the p-Smad2/3 to Smad2/3 ratio **(H)** in each group. **(I)** Immunofluorescence staining showing the expression and subcellular distribution of p-Smad2/3 in CFs; original magnification, ×200; p-Smad2/3 was labeled with red fluorescence. **(J)** Quantitative analysis of the fluorescence intensity of p-Smad2/3 in each group. *P < 0.05 vs. normoxia group; **P < 0.01 vs. normoxia group; ^#^*P* < 0.05 vs. hypoxia group; ^##^*P* < 0.01 vs. hypoxia group.

To further elucidate the crucial role of the glutamatergic transmitter system in mediating collagen synthesis in CFs under hypoxic conditions, CNQX and MK-801 were employed to inhibit iGluRs. Western blot analysis demonstrated that the hypoxia-induced upregulation of Collagen I and Collagen III proteins was significantly attenuated by CNQX and MK-801 (Figures 7A–C). However, neither CNQX nor MK-801 affected the expression of Collagen I and Collagen III proteins in CFs under normoxic conditions. Moreover, hypoxia led to substantial calcium ion accumulation in the cytoplasm of CFs, as evidenced by a significant increase in fluorescence intensity. Notably, both CNQX and MK-801 reduced intracellular calcium levels in hypoxia-stimulated CFs (Figures 7D,E).

Given the well-established role of the TGF-β/Smad signaling pathway in mediating MF (23), we investigated changes in this pathway in CFs under hypoxia. Western blot analysis showed that the TGF-β/Smad signaling pathway was significantly activated in the hypoxia group compared to the normoxia group, as indicated by increased TGF-β protein levels and an elevated ratio of p-Smad2/3 to Smad2/3. Importantly, pretreatment with CNQX and MK-801 almost completely reversed this aberrant activation (Figures 7F–H). Since phosphorylated Smad2/3 can translocate to the nucleus, where they bind DNA to regulate transcription of fibrosis-related genes (23), immunofluorescence staining was performed to visualize the expression and localization of p-Smad2/3. As shown in Figures 7I,J hypoxia markedly increased the fluorescence intensity of p-Smad2/3, which was significantly inhibited by CNQX and MK-801 pretreatment. Collectively, these findings suggested that the regulatory effects of L-glutamate on promoting collagen synthesis in hypoxia-stimulated CFs might be mediated by the TGF-β/Smad signaling pathway.

## Discussion

**Figure 8:**
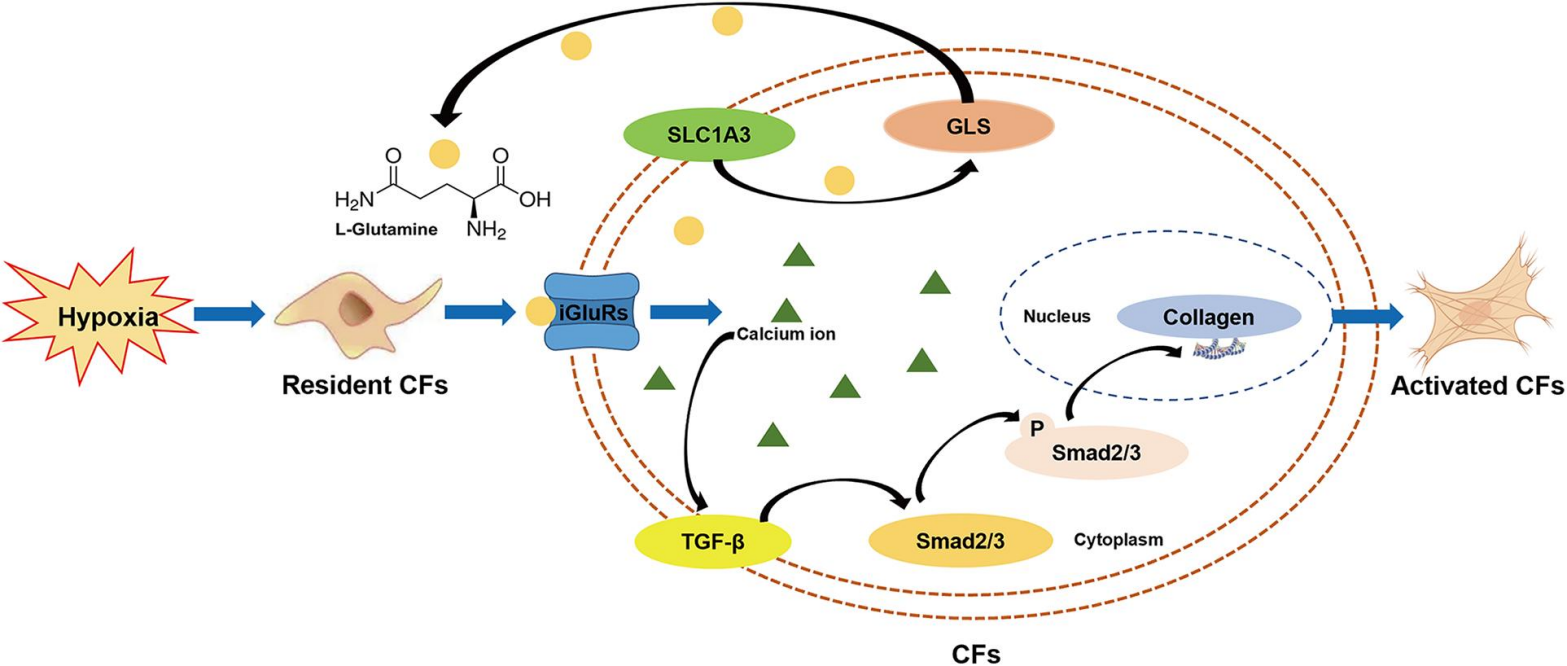
Schema illustrating the potential effects and underlying mechanisms of the glutamatergic transmitter system in mediating CFs activation under hypoxia.

This preclinical study, employing non-targeted metabolomics profiling, provides the first evidence that L-glutamate levels are elevated in the secretions of CFs under hypoxic conditions. Pharmacological blockade of iGluRs effectively inhibits L-glutamate-induced collagen synthesis in CFs through modulation of the TGF-β/Smad signaling pathway. These findings highlight the pivotal role of the glutamatergic transmitter system in mediating CFs activation (Figure 8), providing novel insights into the molecular mechanisms underlying MF.

Hypoxia serves as an important trigger for MF development in multiple pathological conditions, including MI, ischemic heart disease, and pulmonary hypertension (24). Emerging evidence indicates that under hypoxic conditions, HIF-1α can be activated and translocate to the nucleus, where it binds to hypoxia-response elements (HREs) in the promoter regions of target genes such as vascular endothelial growth factor (VEGF), TGF-β, and connective tissue growth factor (CTGF), which are well-known to promote fibroblast activation and ECM production (25). Previous proteomics analyses have confirmed that hypoxia selectively upregulates the expression of ECM-related proteins in the secretory products of CFs (3). Our study revealed significant morphological changes in CFs with prolonged hypoxia exposure, characterized by cell elongation, spindle-shaped transformation, and excessive ECM deposition. Given that collagen is a major component of ECM and its synthesis plays a crucial role in MF progression, we specifically examined Collagen I and Collagen III proteins in CFs. Both were upregulated following hypoxia stimulation. These findings, combined with the observed morphological alterations in CFs, collectively demonstrated that hypoxia could promote collagen synthesis in CFs.

Interestingly, although hypoxia has been reported to induce myofibroblast differentiation in CFs (26, 27), our experiments did not demonstrated significant alterations in α-SMA expression under hypoxia. This discrepancy may be attributed to the stringent experimental protocol during hypoxia exposure. Unlike previous methodologies (28–30), in the present study, CFs were maintained in serum-free medium during hypoxia stimulation to eliminate potential confounding factors. This precaution was taken due to the consideration that various growth factors, hormones, and nutrients in serum could potentially compromise the accuracy and precision of subsequent metabolomics analyses. Therefore, the absence of these serum-derived components may attenuate the hypoxia-induced upregulation of *α*-SMA.

The ability to secrete a variety of substances is an important characteristic of fibroblasts. In addition to the well-known production of collagen, CFs are capable of secreting various cytokines, chemokines, fibronectin, MMPs, TIMPs, and CTGF, all of which contribute to the development and progression of MF (31). Previous research has reported that CFs-derived factors can significantly reduce cardiomyocyte viability (3), indicating a substantial paracrine effect of CFs on altering the myocardial microenvironment. In the present study, non-targeted metabolomics profiling was employed to investigate the metabolic alterations in CFs under hypoxia. Our analysis identified 44 differentially expressed metabolites in the secreted products from hypoxia-stimulated CFs. Among them, L-glutamate levels were significantly elevated, and such elevation was subsequently validated through both *in vitro* and *in vivo* experiments. Furthermore, the differentially expressed metabolites induced by hypoxia were predominantly enriched in the arginine and proline metabolism pathways. L-glutamate, synthesized from *α*-ketoglutarate through the action of glutamate dehydrogenase or GLS, serves as a crucial precursor for the production of glutamine, proline, and arginine (32). Consequently, L-glutamate plays an essential role in regulating the arginine and proline metabolism pathways, thereby maintaining nitrogen balance, energy production, and biosynthesis of critical molecules (33). Although the precise mechanism by which CFs mediate the upregulation of L-glutamate levels remains challenging to elucidate, the observed increase in L-glutamate levels can represent a significant biomarker for CFs activation under hypoxic conditions.

The baseline levels of glutamate have been reported to be positively associated with an elevated risk of cardiovascular disease (34). Plasma metabolic profiling has demonstrated that L-glutamate levels are markedly higher in patients with stable angina pectoris and MI compared to healthy controls, and it can be identified as a core metabolite for diagnosing coronary heart disease (35). This observation is further supported by a study showing increased L-glutamate levels in both myocardial tissues and plasma of MI rats (36), which is consistent with the findings from our animal experiments. Based on this evidence, we detected the key components of the glutamatergic transmitter system in CFs. Although both iGluRs and mGluRs are two major classes of receptors that respond to glutamate, iGluRs function as ligand-gated ion channels facilitating rapid excitatory signaling, while mGluRs predominantly modulate synaptic activity over extended periods (14). Consequently, we examined all iGluR subtypes and identified that only GRIA3, GRIN2, and GRIK5 were relatively highly expressed in CFs. Subsequent RT–PCR analysis demonstrated that hypoxia could upregulate GRIA3 and GRIN2 mRNA levels but did not affect GRIK5 expression. Additionally, SLC1A3 (also known as EAAT1), a glutamate transporter responsible for cellular glutamate uptake (37), along with GLS, an enzyme that catalyzes the conversion of glutamine to glutamate (38), were detected. Our results revealed that SLC1A3, GLS, GRIA3, and GRIN2 were all significantly upregulated in hypoxia-stimulated CFs and myocardial tissues of MI rats. Therefore, these findings collectively indicated that the glutamatergic transmitter system was significantly activated in CFs under hypoxia.

The regulatory effects of the glutamatergic transmitter system on CFs activation were systematically verified. Our results demonstrated that blocking iGluRs with CNQX and MK-801 effectively suppressed hypoxia-induced profibrotic effects on CFs. Notably, L-glutamate promoted collagen synthesis in CFs even under normoxic conditions. Therefore, the glutamatergic transmitter system may serve as a crucial mediator of hypoxia-induced CFs activation. Importantly, glutamate is involved in the malate-aspartate shuttle, which plays an essential role in ATP production in cardiomyocytes (39). Although SLC1A3 expression is significantly lower in rat hearts compared to brain tissue (40), it has been reported to facilitate the metabolic utilization of glutamate, thereby improving ATP supply and cell viability in ischemic myocardial injury (41). Furthermore, pharmacological inhibition of GLS exacerbates cardiac dysfunction in mice with MI (42). These findings further underscore the crucial role of the glutamatergic transmitter system in regulating the progression of cardiovascular disease, particularly ischemic heart disease.

As a multifunctional cytokine, TGF-β plays a pivotal role in regulating cell proliferation, differentiation, and ECM production. Upon binding to its receptor on the cell membrane, Smad2/3 are phosphorylated by TβRI and subsequently translocate into the nucleus, where they function as transcription factors to modulate the transcription of fibrosis-related target genes (23). The canonical TGF-β/Smad signaling pathway is well-established as a critical regulator of MF and cardiac remodeling (43). Hypoxia-inducible factors (HIFs), which are activated under hypoxic conditions, have been shown to mediate the transcriptional activation of TGF-β in various cell types (44–46). Thus, hypoxia serves as a potent inducer of TGF-β signaling. Consistent with previous studies (47, 48), our study revealed that hypoxia significantly activated the TGF-β/Smad signaling pathway in CFs. Notably, blockade of iGluRs almost completely suppressed this hypoxia-induced activation, suggesting a close inner link between the glutamatergic transmitter system and the TGF-β/Smad signaling pathway. Previous evidence has confirmed that glutamate elevates intracellular calcium levels through iGluRs activation, with calcium ion serving as a crucial second messenger in modulating intracellular signaling cascades (49). Consistent with our findings in CFs, a recent study reported that hypoxia enhanced glutamate-induced calcium influx in horizontal cells isolated from goldfish (50). Given the well-established interplay between intracellular calcium dynamics and TGF-β signaling activation (51, 52), it is plausible that the regulatory effects of glutamate on TGF-β/Smad signaling are mediated through calcium-dependent mechanisms. However, the precise molecular mechanisms underlying the involvement of the glutamatergic transmitter system in CFs activation under hypoxia warrant further investigation.

Although L-glutamate has demonstrated substantial profibrotic effects under hypoxic conditions, it is important to note that other metabolites may also play critical roles in mediating this process. Given the complexity and breadth of the metabolic landscape, a comprehensive evaluation of all differentially expressed metabolites exceeds the scope of the current study. To facilitate further research in this area, we have provided the complete raw metabolomics dataset in the supplemental materials, thereby enabling other investigators to conduct in-depth analyses and validation studies. Additionally, more extensive animal experiments are warranted to confirm the key regulatory role of L-glutamate in the progression of MF.

## Conclusion

In conclusion, the present study demonstrates that hypoxia elevates L-glutamate levels in CFs secretions, and that inhibition of iGluRs effectively attenuates hypoxia-induced collagen synthesis through modulation of the TGF-β/Smad signaling pathway. These findings highlight the pivotal role of the glutamatergic transmitter system in hypoxia-induced CFs activation, providing novel insights into the therapeutic strategies for MF.

## Data Availability

The raw data supporting the conclusions of this article have been deposited in the China National Center for Bioinformation database (Accession Number: OMIX012149).

## Supplementary material

Supplementary Figure 1 Cell proliferation and identification of CFs isolated form neonatal rats. (A) The dynamic growth of CFs was monitored at different time points following isolation. (B) Immunofluorescence staining was employed for CFs identification, with vimentin serving as a fibroblast-specific marker (green fluorescence); original magnification ×200.

Supplementary Figure 2 Morphological changes in CFs under hypoxia at various time points. Images were observed and captured using an optical microscope, while corresponding cellular oxygenation levels were assessed using Image-IT™ Green Hypoxia Reagent; original magnification ×100.
