# Supplementary figures and images for: The endogenous glutamatergic transmitter system promotes collagen synthesis in cardiac fibroblasts under hypoxia
Source: Front Cardiovasc Med. 2025 Oct 17;12:1638650. doi: 10.3389/fcvm.2025.1638650 (PMC12575353; doi:10.3389/fcvm.2025.1638650)

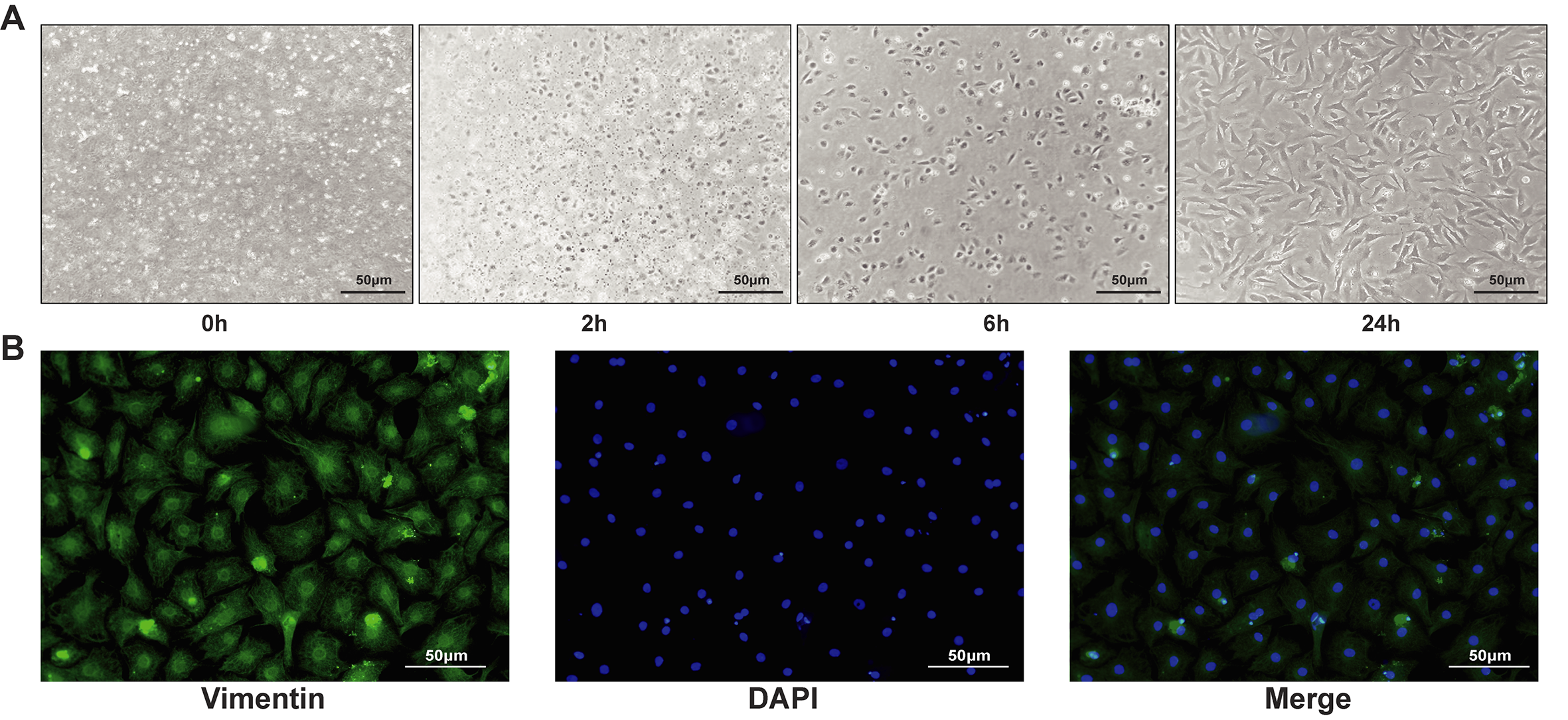

Supplement: Supplementary Figure 1 — Cell proliferation and identification of CFs isolated form neonatal rats. (A) The dynamic growth of CFs was monitored at different time points following isolation. (B) Immunofluorescence staining was employed for CFs identification, with vimentin serving as a fibroblast-specific marker (green fluorescence); original magnification ×200. [file Image1.tif]

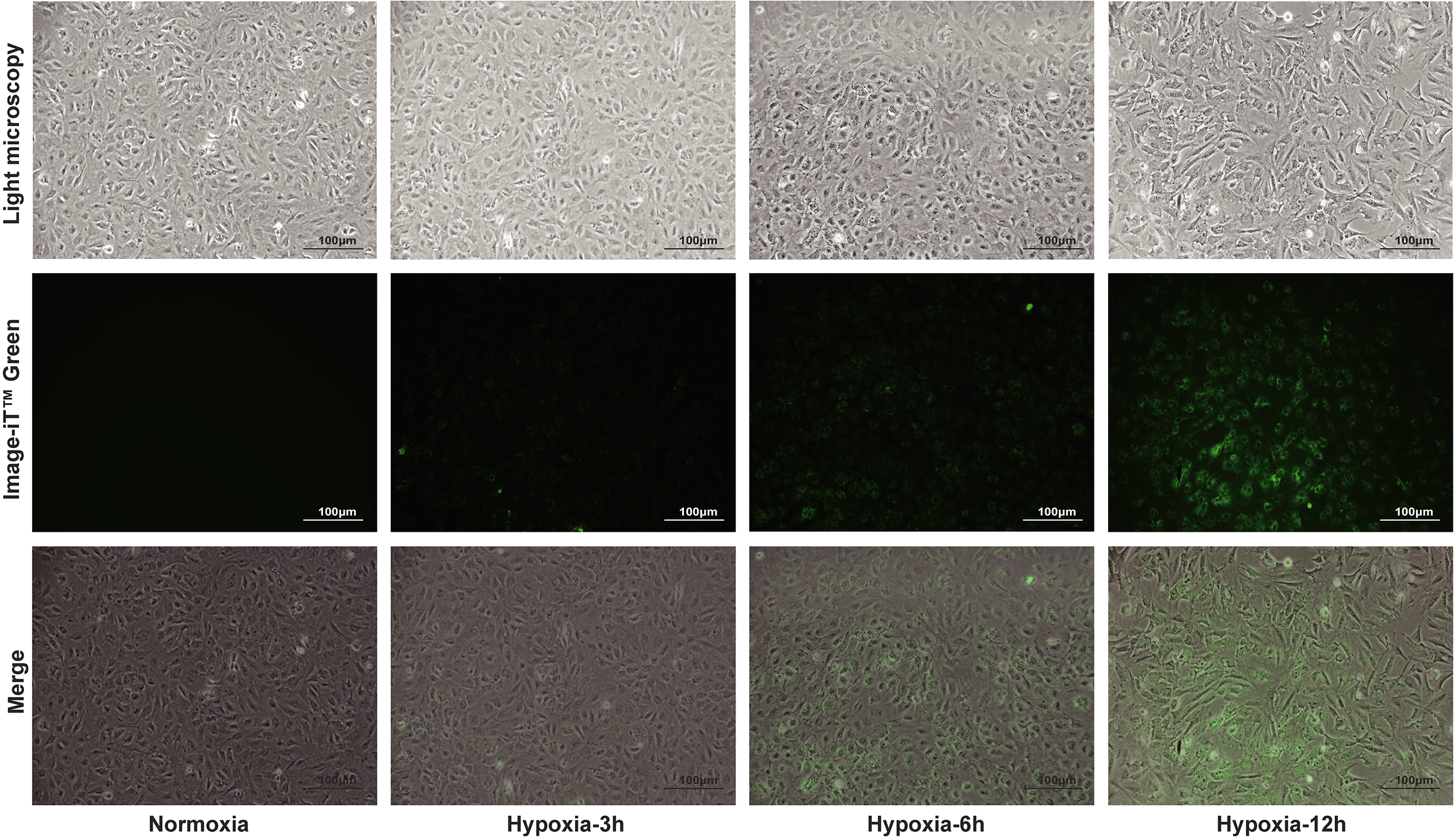

Supplement: Supplementary Figure 2 — Morphological changes in CFs under hypoxia at various time points. Images were observed and captured using an optical microscope, while corresponding cellular oxygenation levels were assessed using Image-IT™ Green Hypoxia Reagent; original magnification ×100. [file Image2.tif]
